# The association of both self-reported and behavioral impulsivity with the annual prevalence of substance use among early adolescents

**DOI:** 10.1186/s13011-015-0019-0

**Published:** 2015-06-10

**Authors:** Víctor Martínez-Loredo, José Ramón Fernández-Hermida, Sergio Fernández-Artamendi, José Luís Carballo, Eduardo García-Cueto, Olaya García-Rodríguez

**Affiliations:** Department of Psychology, Faculty of Psychology, University of Oviedo, Oviedo, 33003 Spain; Department of Psychology, University of Miguel Hernández, Alicante, Spain

**Keywords:** Adolescents, Impulsivity, Substance use, Delay discounting, Disinhibition, Self-reports

## Abstract

**Background:**

In relation to substance use, Spanish adolescents aged 12 to 14 can be largely classified in four groups, from highest to lowest prevalence: a) No substance use, b) Only alcohol use, c) Alcohol and tobacco use, and d) Alcohol, tobacco and cannabis use. The aim of the present study is to analyze the possible relationship between impulsivity and the substance-use group to which the young person belongs

**Methods:**

One thousand three hundred and forty-eight adolescents aged 12 to 14 in northern and eastern Spain reported their drug use, completed impulsivity self-reports (BIS-11-A and ImpSS) and performed behavioral tasks (Stroop Test and Delay Discounting).

**Results:**

Results from both measurement approaches were related to early drug use. An increasing impulsivity trend is found across groups from less to more substance involvement, except in the case of Delay Discounting, which is sensitive only for those with more substance-involved.

**Conclusions:**

Impulsivity is a key factor for early drug use, especially as regards more substance-involved. This should be taken into account in designing prevention programs or as a key variable for interventions aimed at delaying the onset of substance use.

## Background

Age of onset of drug use in Spain, especially alcohol and tobacco, is around 13 years [1]. Among adolescents, the annual substance-use involvement falls within one of four main groups, from less to more involvement: 1) no substance use (NSG), 2) only alcohol use (AG), 3) alcohol and tobacco use (ATG), and 4) alcohol, tobacco and cannabis use (ATCG) [2]. Early substance use is a risk for the health and well-being of adolescents, especially when different substances are mixed [3]. One important research goal is to identify the factors mediating early substance use and, even more, use in high-risk patterns such as the mixed use of different substances. Identifying these factors could improve the effectiveness and efficiency of selective and indicated prevention strategies.

Impulsivity is an important factor related to the onset of drug use [4] and for predicting the escalation of alcohol, tobacco and marijuana use in adolescents [5]. Although there is no agreed definition of impulsivity [6–8], most definitions include features such as lack of planning, inattention, preference for sooner outcomes or lack of capacity to be focus on a task [9, 10]. For its measurement, both psychometric instruments and behavioral tasks can be used [11].

Despite this, most studies have focused on a single substance [12] and a single method for assessing impulsivity [4], and used samples with young adult participants aged 15 upwards [13, 14]. This last point is relevant because at such ages the substance use has already had time to become established, so that the results can be influenced by the bidirectional effect of impulsivity and drugs [15, 16].

To our knowledge, there are only two studies that analyze the relationship between impulsivity and early drug involvement using a non-clinical sample aged around 12. The authors of one of these studies found that behavioral impulsivity, assessed with the Delay Discounting Task, Stop-signal Task and Balloon Analogue Risk Task (BART), significantly predicted alcohol use 6 months later [17]. However, this research was focused on alcohol use. Another study examined only the role of behavioral disinhibition in predicting substance use disorder, pointing out that this construct is a better predictor of substance use disorders than frequency of use [18]. The present study assesses precisely this facet of impulsivity, in addition to others.

The goal of this paper is to study the level of impulsivity, measured by both self-reports and behavioral tasks, and its association with early substance use (alcohol, tobacco and cannabis) in a non-clinical sample of early adolescents. Our hypothesis is that impulsivity will be greater in the more substance-involved group.

## Methods

This is a cross-sectional study carried out in different Spanish secondary schools. Sampling was performed following a random stratified and incidental procedure, taking into account the city or town sizes.

### Participants

Participants were 1730 adolescents (54.2 % males) aged 12 to 14 (*M* = 13.04; S.D = 0.510). They were recruited from a total of 22 secondary schools in two different Spanish regions.

In the sample used for the analysis, having fulfilled all the inclusion criteria, there were 1348 participants (77.92 % of the total). The data reduction procedure is explained in a later section. This study was approved by the Ethics Committee of the Spanish Education Ministry, and no participants refused to be assessed.

### Measures

#### Socio-demographic measures

In the first part of the survey participants were asked to indicate their age and gender.

#### Drug use measures

Alcohol, tobacco and cannabis use over the past year was assessed using items from the European School Survey Project on Alcohol and Other Drugs (ESPAD) [19] for assessing annual prevalence of substance use. The Survey assesses the use of tobacco, alcohol and illegal drugs in the school-going population of 15–16 year-olds across 36 European countries. Participants were also asked to indicate age of onset of alcohol use.

#### Impulsivity measurement

##### Self-reports

The adolescent version of the Barratt Impulsiveness Scale [20] was used. It is composed of 30 items with Likert-type questions in which participants must state how often they perform different behaviors (from rarely/never to almost always/always). The scale has a total score and three sub-scores. As there is no consensus about how many subscales are adequate for adolescents [21, 22], the only score used was the total score proposed by the authors of the questionnaire, which refers to a general trait of personality. The Spanish adaptation [23] showed good reliability and consistency (*α* = .82).

The Impulsive Sensation-Seeking (ImpSS) Scale, part of the Zuckerman-Kuhlman Personality Questionnaire (ZKPQ) [24]. This subscale has 19 true/false (false 0, true 1) items which provide a general score and two sub-scores: impulsivity (Imp) and impulsive sensation-seeking (SS). The scale assesses preference for change and uncertainty, as well as the tendency to act without thinking or planning. A Spanish adaptation [25] was employed, showing good consistency (*α* = .81).

##### Behavioral tasks

Two of the behavioral tasks most widely used for assessing impulsivity (Stroop Test and Delay Discounting) were employed. These tasks assess specific behavioral processes based on normative situations in which participants have to behave without any introspection. They are supposed to measure more state-dependent impulsivity than self-reports, which assess impulsivity traits.

A computerized version of the original Stroop task [26] was designed. Three blocks with 30 stimuli displayed in four colors (blue, green, red and yellow) were included in the task: a first block of neutral stimuli (XXXX) appearing randomly; a second block of congruent stimuli (name and ink color matched); and a third block of incongruent stimuli (name and ink color unmatched). Number of errors was recorded. This instrument conceived impulsivity as difficulties in suppressing competing information in order to maintain response performance.

A Delay Discounting (DD) task was also used [27]. The students made choices between a small sum of hypothetical money available immediately and 1000 euros available after seven different periods of time (1 day, 1 week, 1 month, 6 months, 1 year, 5 years and 25 years). Delay discounting was calculated using Log*k* and the area under the curve (AUC). The AUC measure makes no assumptions about the data, is not linked to any theoretical framework, and ranges between 0 and 1 [28]. Impulsivity is defined by DD as the tendency to choose smaller, relatively immediate rewards over larger but more delayed rewards.

#### Control variables

With the aim of detecting random answers, an infrequency scale was used (Oviedo Infrequency Scale, INF-OV) [29]. This instrument is composed of 12 items mixed throughout the assessment. Participants are required to respond to Likert-type items (from totally disagree to totally agree) about obvious facts such as ‘I know people who wear glasses’ or ‘I have sometimes watched films on TV’. Participants with more than three points on the scales were removed.

#### Procedure

All the questionnaires and behavioral tasks used were computerized versions of traditional paper-and-pencil surveys, adapted to an electronic tablet framework (Samsung Galaxy Tab2 10.1). This method was used with the aim of reducing inconsistent answers. The software did not allow participants to skip any questions and was designed to avoid asking inappropriate questions in accordance with previous answers.

#### Data reduction and analysis

**Fig. 1:**
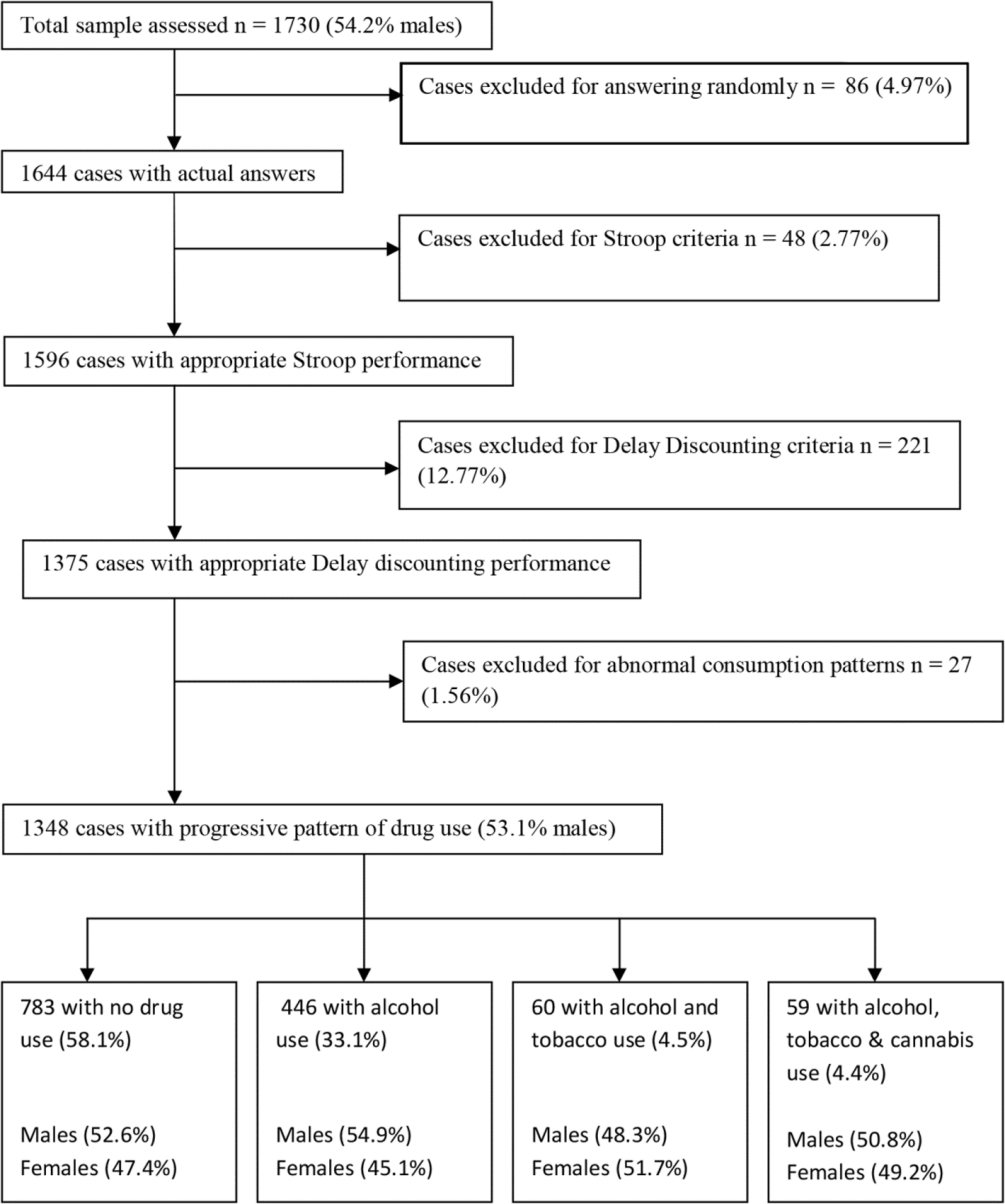
Flowchart of sample progression. Legend: number and percentage of participants removed for each criterion is shown. Final groups are described by number and percentage of participants as well as percentage of males and females in each group

To clean up the sample and remove respondents with invalid answers we followed the steps set out in Fig. 1. In this study, a case is considered valid only when valid answers are obtained in all the questionnaires and tasks, following the rules established for each instrument.

Eighty-six cases had three or more infrequent answers in INF-OV, and were removed following the rule established by the authors.

In the Stroop test, participants with outlier reaction times (defined as faster than 200 ms or slower than 2000 ms, and then if they were more than 3 SD above the participant’s mean RT) were removed, following Fernie et al. [17].

For the DD tasks, respondents who answered irrationally were also removed. For this purpose we used an algorithm based on that of a previous study [30], but with some modifications: 1) if there is more than one indifference point greater than the preceding point by a magnitude greater than or equal to 20 % of the larger later reward, or 2) if the last indifference point was not less than the first indifference point by at least a magnitude equal to 10 % of the larger later reward. Scores in AUC were reversed in order to be interpreted in the same way as the other measures.

Twenty-seven participants had a substance-use pattern that precluded them from classification in any of the established groups. They were therefore removed from the analysis.

To test the hypotheses and due to the lack of normality of the distribution of some variables, a Kruskal-Wallis test for k-independent samples was performed for each test with a post-hoc test between groups, based on the Bonferroni inequality [31]. Confidence level for multiple comparisons was established at 95 %. The effect size was calculated with Grissom and Kim’s estimator [32], interpreted with Cohen’s criteria. This effect size measure yields the probability that a randomly chosen participant in a group with more substance involvement will have a higher score than a randomly chosen participant from another group with less substance involvement [33]. A Jonckheere-Terpstra test was also performed to test trends of increased impulsivity across the groups, independently of statistically median differences between groups.

For variables with normal distribution, a one-way between-groups analysis of variance was conducted with a Tukey HSD post-hoc test. Effect size was calculated using Cohen’s d. Reliability of self-reports BIS-11-A and ImpSS were *α* = .91 and *α* = .83, respectively.

## Results

The Kolmogorov-Smirnov test was performed to assess the normality of the distributions and sex differences in Impulsivity scores were also assessed due to their possible influence. Results of both analyses are shown in Table 1. Because of the statistical significance of sex differences in BIS and Stroop errors separate tests were performed for males and females in both measures.

**Table 1:** Distribution of variables of the overall sample and males and females scores comparison

Variable	Total (*n* = 1348)	Males (*n* = 716)	Females (*n* = 632)	*p*-value
BIS-11-A (median)	39^**^	37	40	.005^†^
ImpSS (median)	9^**^	9	9	.457^†^
Stroop (median)	1^**^	1	1	.001^†^
AUC (median)	.1549^**^	0.1582	0.1504	.925^†^
Log K (mean ± SD)	−2.19 ± 1.10	−2.22 ± 1.10	−2.15 ± 1.11	.281^‡^

^**^ *p* ≤ .001 significant level in Kolmogorov-Smirnov normality test; p-value column corresponds to comparisons between males and females; ^†^ Mann–Whitney test; ^‡^ *t*-test; SD standard deviation; BIS: Barrat Impulsivity Scale; ImpSS: Impulsive Sensation Seeking; Stroop: number of errors in Stroop test; AUC: Area Under the Curve in Delay Discounting

The Kruskal-Wallis test showed statistically significant differences in self-report scores between substance-involvement groups for ImpSS (*χ*^2^ (3) = 171.921, *p* < .05) and also for BIS-A in both sexes (*χ*^2^ (3) = 75.598, *p* < .00 for males; *χ*^2^ (3) = 85.140, *p* < .00 for females).

After the post-hoc comparison, all groups showed significant differences in both self-reports, with the exception of ATG compared to ATCG on Zuckerman’s ImpSS scale. In all cases, a higher score on the scale was related to greater substance involvement (see Table 2). Effect sizes were moderate, ranging between 0.19 and 0.39 for BIS for both sexes and between 0.21 and 0.35 for ImpSS.

**Table 2:** Post-hoc comparisons of impulsivity measures in Kruskal-Wallis test among annual prevalence of substance-involvement groups

	Self-reports			Delay-Discounting
Group	BIS-11-A		ImpSS	AUC
	males	females		
NSG				
Median	57 ^a^	59^a^	8 ^a^	154 ^a^
AG				
Median	61 ^b^	64^b^	10 ^b^	168 ^a^
ATG				
Median	70 ^c^	73^c^	14 ^c^	154 ^a^
ATCG				
Median	74 ^d^	81 ^d^	15 ^c^	.074 ^b^

Note. Mann–Whitney post-hoc test was performed to assess the median differences between groups. Same letter means no difference between groups and different letter means differences between groups. All comparisons in BIS, ImpSS and comparisons in AUC between AG and ATCG were significant at .001 level. Comparisons in AUC between NSG and ATCG, and ATG and ATCG were significant at .05 level

As regards the Stroop Test, the Kruskal-Wallis test showed no significant differences in the number of errors between groups in males (*χ*^2^ (3) = 6.041, *p* = .11) or in females (*χ*^2^ (3) = 7.14, *p* = .068.

As far as Delay Discounting was concerned, the analysis showed statistically significant differences between groups for the AUC (*χ*^2^ (3) = 11.461, *p* < .05).

Visual inspection of the AUC ranks indicated three comparisons (NSG-ATCG, AG-ATCG and ATG-ATCG). Significant differences were found for the three comparisons (see Table 1). The effect sizes were, respectively, 0.38, 0.37 and 0.36.

**Fig. 2:**
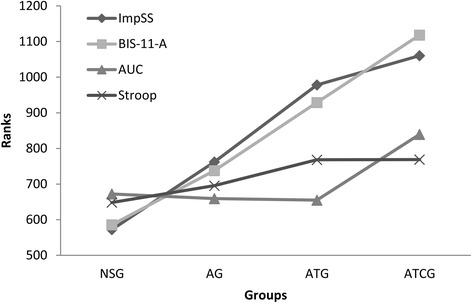
Impulsivity level trends among annual prevalence of substance-involvement groups. Legend: y-axis shows mean ranks in Kruskal-Wallis test for all Jonckheere-Terpstra test significant measures. X-axis shows the different substance-use groups

The results of the Jonckheere-Terpstra test were significant for both the BIS in males (J-T = 7.63, *p* < .001, *r* = .29) and females (J-T = 8.81, *p* < .001, *r* = .35) and the ImpSS (J-T = 12.73, *p* < .001, *r* = .35). With respect to behavioral tasks, the DD discounting did not yield significant results (J-T = 2.28, *p* < .05), but it did do so for number of errors on the Stroop in both males (J-T = 7.63, *p* < .001, *r* = .08) and females (J-T = 2.57, *p* < .05, *r* = .10). As this test shows, although differences between some groups were not statistically significant, an increasing trend of impulsivity was present for the four groups in all the instruments, except in the case of DD (see Fig. 2).

To explore the impact of Log*k* score on Delay Discounting, a one-way between-groups analysis of variance (ANOVA) was carried out. There was a statistically significant difference (*p* < .05) between groups: F (3, 1344) = 3.392, *p* = .017. Post-hoc comparison using the Tukey HSD test indicated that the mean scores for NSG (*M* = −2.19, SD = 1.12) and AG (*M* = −2.24, SD = 1.08) were significantly different from ATCG scores (*M* = −1.76, SD = 1.1). The effect sizes were 0.39 and 0.44, respectively, in the same direction as the study’s hypothesis.

Mean age of onset of alcohol use was 11.53 years (S.D. = 1.55), and there were no statistically significant differences between groups AG, ATG and ATCG (*χ*^2^ (2) = 0.86, *p* = .651). Thus, the increased impulsivity across the groups cannot depend on the difference in use onset, nor probably on the substances used, since they have been used for only a short time.

## Discussion

In this research the relationship between impulsivity and early substance use was explored with a multimethod perspective among adolescents aged 12 to 14. In our data we found that there is a clear difference in impulsivity according to both self-reports and behavioral tasks in adolescents with early onset of substance use. Furthermore, groups of adolescents with more substance involvement in the last year tend to show higher levels of impulsivity than those with less substance involvement.

This finding is consistent with previous results [4, 14]. However, in comparison to these studies, which use older samples, this research shows for the first time the difference in impulsivity among very early adolescents in relation with their substance involvement. Progression from less to more substance involvement is statistically significant in self-reports. In the literature, the association between sensation-seeking assessed by the ImpSS and the use of cannabis is not consistent. While some studies, both cross-sectional and longitudinal, among adolescents aged 15 found no association [34, 5], other cross-sectional studies among adolescents aged 14–16 did find such an association [35, 36]. This may be explained by the greater influence of other impulsivity sub-traits, as in the case of young adults [37], but more research is needed to clarify the relationship between sensation-seeking and cannabis use among adolescents.

Stroop Test results did not differ between participants who belonged to different groups, though an increasing trend is present in the number of errors committed. Previous studies have shown inhibition problems in heavy-drinking adolescents [12], but these results reflect that inhibition problems can be detected even with low rates of use but more substance-use involvement. This lack of differences among groups despite the trend could have several explanations. A first one might be that prepotent response inhibition does not have very relevant predictive power for early onset of substance use [18]. Another possible explanation is that greater influence of substance involvement appears with more frequent use or is related to the impairment produced by frequent use of substances [38]. From a methodological point of view, it may also be that the digital version of the Stroop Test does not produce the interference phenomenon, making it unsuitable for testing prepotent inhibition, as other studies have shown [39]. However, this last rationale would not explain the increasing trend found.

On the other hand, Delay Discounting AUC and Log*k* were less sensitive to variation in young people’s substance use, but did detect cannabis use. This suggests that adolescents who have more problems to delay gratification are prone to be more substance-use involved, already at these ages. This study, then, provides further evidence of the relationship between DD and cannabis use [40]. The increased level of impulsivity in DD found among early users of cannabis goes in the same direction as the relationship between discounting rates and age at first use found by Heinz [41].

As previous studies show, there is no correlation (or it is very low) between self-reports and behavioral tasks [42, 7]. This lack of relationship, due to the trait/state characteristic of both self-reports and behavioral tasks, could partly explain the different results across the groups. Nevertheless, the increasing trend was found in both types of measure, which may mean that adolescents who already use drugs see themselves as more impulsive than their peers (self-reports), even though they do not behave significantly differently when performing a standardized task (behavioral tasks).

Early onset of alcohol and other substance use increases the risk of having high-risk patterns in adulthood such as the mixed use of different substances. Identifying risk factors associated with this early onset may serve to improve the impact of prevention strategies and interventions aimed at delaying the beginning of substance use [43]. This study provides more evidence about the relevance of impulsivity among early adolescents and its higher level depending on the substances used and the instruments employed to detect it. As stated in previous studies, not only could early identification of impulsive subjects facilitate psychiatric diagnosis, but this information could also be used to monitor those subjects without psychiatric disorders for preventing the early use and possible abuse of substances [14].

The relatively small size of the sample of tobacco and cannabis users in comparison with the other groups is one of the main limitations of this study, but this is an aspect that is difficult to address even in future studies, given the age of the target population: greater use of tobacco and cannabis among adolescents aged 12–14 would not be expected. The absence of measures with regard to possible mediators that could provide more information is another limitation. Furthermore, our cross-sectional design precludes the drawing of causal inferences, so that future research might consider the use of longitudinal assessment of the influence of impulsivity on drug involvement and vice versa, in the style of Fernie et al.’s [17] work.

Despite its limitations, this study analyzed a large sample, which was sufficient to cover different levels of substance-use prevalence, with a multi-method assessment of impulsivity, including self-reports and behavioral tasks for two of the main components (prepotent response inhibition and delay discounting - decision-making) in a digital framework, which prevents errors of transcription. The age group of our participants covered the period when substance use begins, so that even using a cross-sectional design the possible substance-use effect could be attenuated. As Malmberg states, referring to a similar topic [44], it seems plausible to assume that personality (e.g., impulsivity) precedes substance-use behaviors when one assesses a group of early adolescents in the beginning phase of their substance use. In any case, this assertion would have to be confirmed by a longitudinal study. Finally, a strong point of this study is that it assessed the use of not only alcohol but also tobacco and cannabis.

## Conclusions

In sum, the present study shows the presence of higher impulsivity in adolescents with early onset of substance use and its relationship with the level of substance-use involvement. Also, it shows the difference in sensitivity between the self-report tests and the behavioral tests for measuring impulsivity, and how already at these young ages, greater impulsivity is present in people with more substance involvement. These findings have implications for the design of selective and indicated prevention strategies and interventions focused on delaying the onset of substance use, showing the important role of impulsivity in substance use and how different measures indicate disparate sensitivity across different patterns of use.
